# Intra-arterial Administration of Radiopharmaceuticals in Neuro-Oncology; New Improvement for [^131^I]-Phenylalanine in High-Grade Gliomas?

**DOI:** 10.1007/s00270-025-04052-4

**Published:** 2025-05-21

**Authors:** N. Tolboom, T. J. Snijders, E. P. A. Vonken, I. C. van der Schaaf, T. Seute, A. J. A. T. Braat

**Affiliations:** 1 Department of Radiology and Nuclear Medicine, University Medical Center Utrecht, Heidelberglaan 100, 3508 GA Utrecht, The Netherlands; 2 Department of Neurology, Brain Center Rudolf Magnus, University Medical Center Utrecht, Utrecht, The Netherlands

High-grade (WHO grade 3 and 4) gliomas have a poor prognosis (< 20% 5-year survival). The cornerstone of treatment is surgical resection or biopsy followed by external beam radiation therapy, combined with and/or followed by chemotherapy (temozolomide). Once disease recurrence is detected, chances on long-term palliation are slim. To this end, many new drugs and strategies are being designed and explored in this difficult to treat disease. On top of conventional challenges, the blood–brain barrier (BBB) and blood–tumor barrier (BTB) is an additional physical defense, limiting drug extravasation into the brain and toward the tumor. The ‘theranostics concept’ allows radiopharmaceuticals to act as a diagnostics as well as a therapeutic. Within interventional oncology, this is a well-known concept for transarterial radioembolization. Within neuro-oncology/nuclear medicine, a promising theranostic is [^18^F]F-ethyltyrosine ([^18^F]FET; diagnostic) with the therapeutic counterpart [^131^I]I-phenyl-alanine ([^131^I]IPA; TLX101, Telix Pharmaceuticals) [1, 2]. These carriers target the large-amino acid transporter type 1 (LAT-1) and are known to pass the BBB. In its current development, [^131^I]IPA is under investigation in phase 2 studies (NCT03849105 & NCT05450744). Previously, intra-arterial administration of [^177^Lu]Lu-HA-DOTATATE in treatment refractory meningioma patients led to a fourfold increase in tumor uptake [3, 4]. Therefore in the current case, both intravenous and intra-arterial administration of [^131^I]IPA monotherapy was performed, in hopes to boost radiation absorbed dose to the tumor and improve efficacy.

A 41-year-old male presented with a progressive treatment refractory oligodendroglioma (WHO grade 3, 1p19q co-deletion and IDH mutant) in the right parietal lobe. Patient had an extensive medical history (supplemental materials). His last treatment was a debulking resection of a progressive tumor deposition anteriorly in the old resection cavity (Fig. 1A). Postoperative MRI three days after this resection showed a faint linear enhancement (Fig. 1B) and three months thereafter a local recurrence can be acknowledged (Fig. 1C). At this time, a [^18^F]FET-PET was acquired to evaluate the extent of disease (Fig. 1D) showing extensive uptake on the anterior and lateral side of the old resection cavity. As all regular local and systemic treatments were exhausted, a treatment with [^131^I]IPA (TLX101; Telix Pharmaceuticals, Australia) as monotherapy was initiated.

**Fig. 1:**
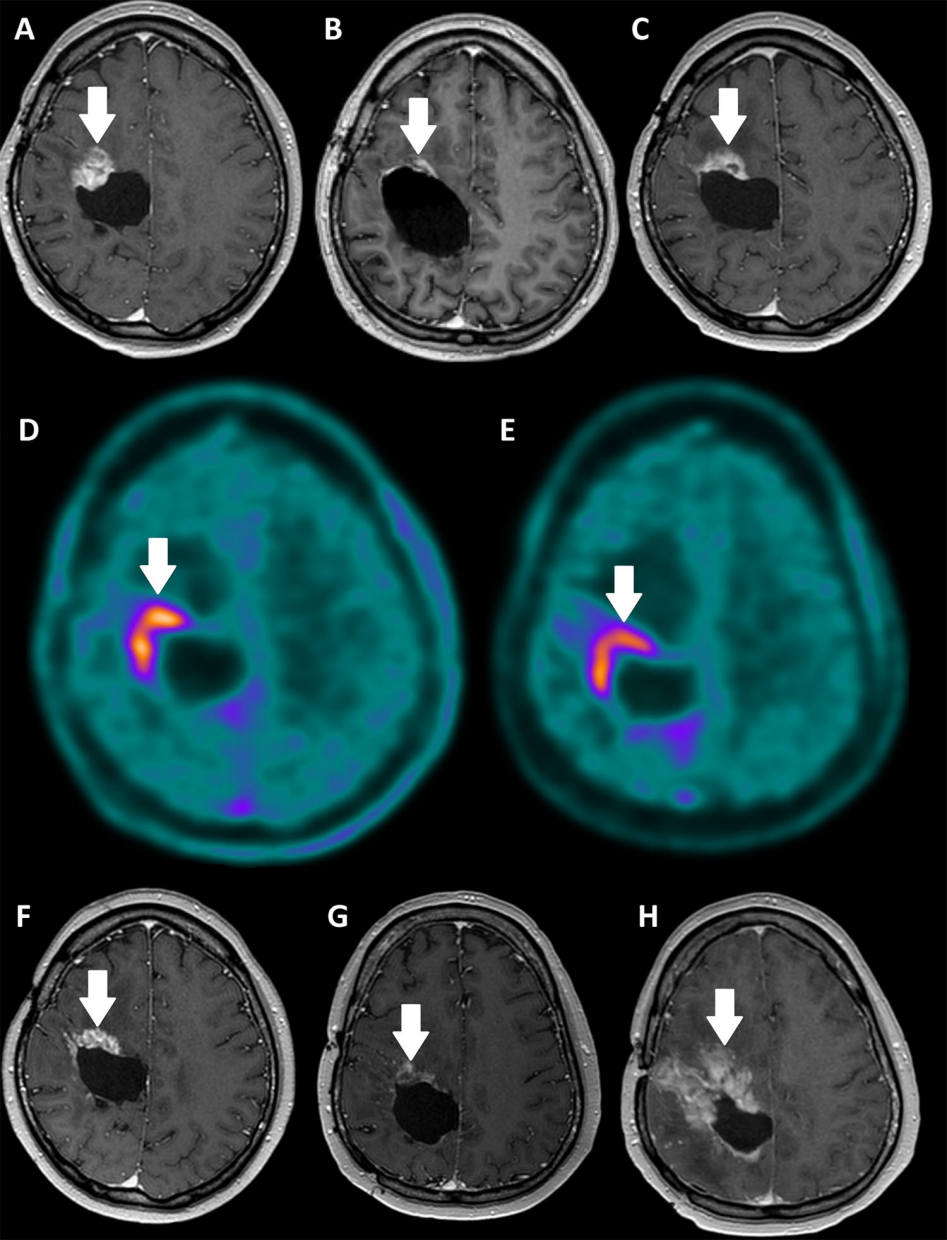
**A** Recurrent anaplastic oligodentritic astrocytoma, WHO grade 3, on a gadolinium-enhanced T1 (gdMR) sequence, shown as an enhancing mass on the anterior side of the primary resection cavity. **B** First postoperative gdMR 4 days after surgical re-resection, showing postoperative rim contrast enhancement in the operation cavity. **C** Follow-up gdMR 3 months after surgical re-resection, demonstrating a recurrent mass in the same location. **D** Flour-18-ethyltyrosine ([^18^F]FET) PET/CT demonstrating pathological uptake (tumor-to-background ratio of 3.2), confirming tumor recurrence and demonstrating patients eligibility for treatment with iodine-131-phenylalanine ([^131^I]IPA)–Theranostic approach. **E** [^18^F]FET-PET/CT 3 months after two cycles of [^131^I]IPA (once intravenous and once intra-arterial), demonstrating stable disease according to PET RANO criteria, but with minor decrease in [^18^F]FET uptake. **F** gdMR 3 months after two cycles of [^131^I]IPA, demonstrating decreased contrast enhancement, stable disease according to RANO criteria. Because of clinical deterioration, at multidisciplinary tumor board (MDT), remaining contrast enhancement on gdMR was deemed to be partially radiation necrosis, for which bevacizumab treatment was initiated. **G** gdMR after two cycles of bevacizumab, demonstrating near complete response with small areas of contrast enhancement remaining. Thus, MDT deemed the interim diagnosis of radiation necrosis valid. H) Multifocal progressive disease nearly 12 months after the two [^131^I]IPA treatments, with clear progression at the same location and new lesions in-cerebro (new lesions not shown). One month later, 13 months after the second cycle of [^131^I]IPA, patient died

Prior to his first treatment with [^131^I]IPA, movement of the left arm and left leg was impaired without other symptoms. The first treatment was performed via intravenous administration of 5 GBq [^131^I]IPA. On posttreatment multiple time point SPECT/CT imaging at 4, 24 and 72 h post-injection (p.i.), a good uptake was seen and a calculated absorbed dose of 2.5 Gy. The patient experienced no additional complaints.

The second treatment performed 21 days later was administered intra-arterial. As there were multiple tumor feeding vessels, administration was done in the right internal carotid artery (Supplemental movie 1). Besides transient dysarthria during administration (10 min), no other complications occurred. On posttreatment multiple time point SPECT/CT, again good uptake was seen and an absorbed dose calculated of 3.2 Gy. The largest increase was at 4 h p.i. of 80%, while 24 and 72 h p.i. increase was 10 and 5%, respectively. During this second hospital admission, complaints of dysarthria recurred. As there were no signs of increased edema or bleeding on an additional CT, steroids were increased, resulting in a partial recovery. After discharge, complete resolution of dysarthria occurred after 3 weeks, but complaints of fatigue persisted.

MRI and [^18^F]FET-PET six weeks after treatment showed stable disease, with limited decreased accumulation of [^18^F]FET (− 10%) and decreased gadolinium enhancement (Figs. 1E, F). MRI 2.5 months after the second treatment showed stable disease and surrounding radiation effects (e.g., radiation necrosis). Increasing symptoms were thought to be caused by the increased radiation effects following treatment, for which bevacizumab treatment was initiated and symptoms stabilized. Unfortunately, after the third cycle of bevacizumab, patient developed extensive central and peripheral pulmonary embolisms and treatment was stopped. A new MRI showed decrease of radiation-induced enhancement following bevacizumab treatment (Fig. 1G) and no new lesions. No new treatments were initiated. One year after the [^131^I]IPA treatment, rapid clinical deterioration occurred, extensive progressive disease was seen on imaging (Fig. 1H) and 13 months after treatment the patient died.

This case confirms the potential of [^131^I]IPA as a monotreatment in a salvage setting of a rapidly progressive [^18^F]FET-positive high-grade glioma [1]. Based on preclinical data, combination of [^131^I]IPA with external beam radiation therapy (EBRT) seems more beneficial, for which the IPAX-1 study was completed, demonstrating safety [2, 5, 6]. Based on our favorable experiences with intra-arterial administration of [^177^Lu]Lu-DOTATATE in meningiomas, we opted for an intra-arterial administration, as additional EBRT was not an option in this patient. Even though this resulted in slight increased tumor absorbed dose, washout of [^131^I]IPA rapidly occurred [3, 7, 8]. Proper prospective studies are needed to define the additional benefit of intra-arterial administration over conventional intravenous administration of [^131^I]IPA and its role with regard to combination therapy with external beam radiation therapy or chemotherapy (temozolomide or lomustine). Based on this initial experience and in the development of this compound, a phase 1 dose escalation study will be initiated in 2026 on intra-arterial administration with astatine-211-phenylalanine ([^211^At]APA), an alpha-emitting radiopharmaceutical, in recurrent glioblastoma.

## Supplementary Information

Below is the link to the electronic supplementary material.

Supplementary file1 (DOCX 13 KB)

Supplementary file2 (AVI 23081 KB)
