# Supplementary material for: Intra-arterial Administration of Radiopharmaceuticals in Neuro-Oncology; New Improvement for [131I]-Phenylalanine in High-Grade Gliomas?
Source: Cardiovasc Intervent Radiol. 2025 May 21;48(6):725–8. doi: 10.1007/s00270-025-04052-4 (PMC12170778; doi:10.1007/s00270-025-04052-4)
Supplement: Supplementary file 1 — Supplementary file1 (DOCX 13 KB) [file 270_2025_4052_MOESM1_ESM.docx]

**Supplemental data**

**Supplemental movie 1.** Lateral digital substraction angiography with the microcatheter in the right internal carotid artery (marked as “ACI re”), showing several tumor feeding vessels arising from the internal carotid and subsequently enhancement of the tumor is seen as a contrast blush.

**Supplemental material. Additional case description**

Additional details on the medical history: patient experienced an epileptic seizure in 2006 based on a low grade glioma, which was eventually resected in 2011 after wait-and-see. Histopathology confirmed a WHO grade 2 oligo-astrocytoma. In 2013 experienced the first recurrence, biopsy proven, WHO grade 2 oligo-astrocytoma for which external beam radiation therapy (cumulative 50 Gy). In 2016 a second recurrence treated with chemotherapy (temozolomide 12 cycles). In 2018, presented again with a recurrence, however now evolved to a WHO grade III anaplastic oligodendroglioma, for which two neurosurgical resections, two types of chemotherapy (re-challenge temozolomide monotherapy and procarbazine+lomustine+vincristine) and re-irradition with external beam radiation therapy (cumulative 35 Gy), all in the course of three years.
